# Automated detection of selected tea leaf diseases in Bangladesh with convolutional neural network

**DOI:** 10.1038/s41598-024-62058-3

**Published:** 2024-06-18

**Authors:** Hafijur Rahman, Iftekhar Ahmad, Parvej Hasan Jon, Abdus Salam, Md. Forhad Rabbi

**Affiliations:** 1 Department of Food Engineering and Tea Technology, Shahjalal University of Science and Technology, Sylhet, Bangladesh; 2 Department of Computer Science and Engineering, Shahjalal University of Science and Technology, Sylhet, Bangladesh

**Keywords:** CNN, Deep learning, Image processing, Tea leaf disease, Engineering, Mathematics and computing

## Abstract

Globally, tea production and its quality fundamentally depend on tea leaves, which are susceptible to invasion by pathogenic organisms. Precise and early-stage identification of plant foliage diseases is a key element in preventing and controlling the spreading of diseases that hinder yield and quality. Image processing techniques are a sophisticated tool that is rapidly gaining traction in the agricultural sector for the detection of a wide range of diseases with excellent accuracy. This study focuses on a pragmatic approach for automatically detecting selected tea foliage diseases based on convolutional neural network (CNN). A large dataset of 3330 images has been created by collecting samples from different regions of Sylhet division, the tea capital of Bangladesh. The proposed CNN model is developed based on tea leaves affected by red rust, brown blight, grey blight, and healthy leaves. Afterward, the model’s prediction was validated with laboratory tests that included microbial culture media and microscopic analysis. The accuracy of this model was found to be 96.65%. Chiefly, the proposed model was developed in the context of the Bangladesh tea industry.

## Introduction

Bangladesh is one of the major tea-producing countries in the world, and approximately 45% of the cultivable grant area is used for tea production^1^. Due to its sophisticated aromatic features, tea finds its place as one of the most widely consumed beverages in the world. Tea provides antioxidant, antibacterial, antiviral, anti-inflammatory, anticarcinogenic properties and reduces the risk of diabetes, obesity, asthma, pancreatitis, and so on^2^.

Disease in plants is an inherent factor of consideration in agricultural production, and it causes a substantial cutback on the overall yield. Diseases are a constraint on the typical growth pattern, consequently altering its critical functions such as pollination, fertilization, transpiration, photosynthesis, germination, etc. Naturally, there will be a periodic spread of disease, and it will be tantamount to a great penalty on overall production if no countermeasures are taken^3^. Tea leaves are often infected by pathogens such as *Cephaleuros spp., Pestalotia spp., Collecotrichum spp.,* etc., which results in tea diseases like red rust, grey blight, and brown blight, respectively. Due to changes in certain predisposing factors such as soil status, pH of the soil, shade, and drainage conditions, pathogens can conveniently penetrate and spread plantations^4,5^. Polycyclic fungal infections such as brown blight, grey blight, and blister blight can cause huge losses under favorable conditions. Data available for South-Asian countries suggests that yield loss estimates range from 20 to 50%^6–9^. Especially red rust is a major widespread disease for tea leaves under detrimental conditions of soil and climate. *Cephaleuros* produces orange or red colored fructifications containing a huge number of spores in infected leaves^10^. Spores are usually miniscule and can easily transmit over long distances, spreading disease along with them^11^. Therefore, early-stage detection of disease in tea plants is necessary to minimize yield loss. Since tea cultivation is carried out on vast acres of land and most of the primary symptoms of tea diseases are microscopic, that’s why the detection of disease is difficult and non-pragmatic for human visual capabilities^12^. Traditionally, plant diseases were explored manually by specialists in the field and required high processing time and laboratory capabilities. Whereas the cultivation and production of quality tea are highly technical, it is undeniable that diseases are identified by the naked eye method to this day^13^. This method is too expensive, requires continuous monitoring via experts, and is also subject to inaccuracy. For effective detection of plant disease, it will prove advantageous to properly utilize technologies and machinery. Image processing techniques are compatible with the detection of different types of disease in the agriculture sector, and they are gathering an increasing amount of attention. The leaf of the plant is considered for its color, texture, shape, and so on^14^.

CNN is a highly popular method to analyze the results and classify the problems in agriculture that merges the approaches of engineering technology and mathematics. The present research work has been undertaken to develop a deep learning model based on CNN for the automated detection of selected tea leaf diseases in Bangladesh and pave the way for future prospects for a wider range of applications. A dataset of tea leaves has been created by collecting samples from several tea fields to implement the proposed model. The dataset is used to detect four classes of leaves, namely grey blight, brown blight, red rust, and healthy leaf, and aims to provide an effective solution for identifying common tea foliage diseases in Bangladesh.

## Literature review

Plant parts are diseased when microorganisms such as fungi, bacteria, and viruses invade a plant to thwart its natural growth^15^. The disease of tea can affect several parts, such as the leaf, stem, nods, and root. In this work, only selected major tea diseases of Bangladesh are considered relevant to tea leaves, including (a) ‘brown blight’ disease, one of the destructive foliage diseases of tea-producing countries. The disease is caused by *Colletotrichum camelliae*, which is widely regarded as the most significant plant pathogenic fungus worldwide^16^. Isolation and identification of varied characteristics and pathogenicity of *Colletotrichum camelliae and Colletotrichum fructiocola* from various tea plants were done^17^, where they collected 38 *Colletotrichum* from tea plants and cultured the pathogens on PDA and SNA medium for the collection of conidia and appressoria. (b) ‘grey blight/blister blight’ disease is also a destructive, widespread fungal disease. The disease is also caused by *Exobasidium vexans,* which affects production in several tea-dependent economies, resulting in huge losses. This disease generally impacts tea crops from June to September^18^. (c) ‘red rust’ disease is an algal disease that can infect tea plants. The algae produce huge spores, which are hypha-like vegetative bodies. After passing the maturation stage, the sporangia are separated and spread around by rain, dew, and wind to healthy plants. Young and old tea plants are attacked via red rust under unfavorable conditions of soil and climate^19^. Cultural characteristics of *Cephaleuros parasiticus* and a microscopic view of zoosporangium-containing zoospores were presented by^10^. They curated tea leaves infected by red rust disease from several tea plantations in southern India. They tested the different broth media for isolation and found that Trebouxia and Bristol media were most suitable for *C. parasiticus.*

One study evaluated seven types of tea leaf diseases using an artificial neural network. For classification, they got 90.16% accuracy for LeafNet, superior to conventional SVM and MLP algorithms^20^. Another similar study generated a method to identify and classify plant leaf diseases via k-means clustering and artificial neural networks with a precision of around 93%^21^. Leveraging the computational efficiency of MobileNet, researchers investigated its potential for robust plant leaf disease detection and classification tasks. They worked on 5 categories of tomato disease and used 5512 images while obtaining 98.7% average accuracy^22^. MobileNet was also employed for disease detection in cassava plants in another study where researchers obtained an impressive precision of 80.6% on a dataset of images and 70.4% on a dataset of videos^23^. Researchers used both AlexNet and VGG16 to classify tomato crop diseases. They got a classification accuracy of 97.29% for VGG16 and 97.49% for AlexNet from the image dataset obtained from a secondary source^24^. One particular report analyzed 1796 images of maize leaves and achieved 97.8% accuracy on the validation set^25^. Another particular model of CNN detected the disease of the tomato plant leaf, with a 98% success rate for VGG-16 and 99.23% for GoogLeNet^26^.

Existing studies on automated tea leaf disease detection using complex models such as ResNet, VGG16, and others require a vast amount of data to achieve an optimal result, which is typically countered with secondary data. This study aims to use high-resolution but limited images collected directly from tea fields in Sylhet, Bangladesh, and achieve a promising result. The contribution of this study is as follows:

1. This work introduces a novel dataset of diseased tea leaf images acquired directly from tea gardens in Sylhet, Bangladesh. This unique dataset can serve as a valuable resource for training and testing CNN models and their modifications for tea leaf disease detection, potentially benefiting both our present study and future research efforts in this domain.
2. To our knowledge, limited research was conducted with CNN to recognize patterns of brown blight, grey blight, and red rust along with healthy leaves from primary images. This study shows the implementation of a basic CNN architecture to achieve competitive accuracy.
3. This study, for the first time, uses an additional layer of validation testing where an independent set of diseased and healthy leaves collected from fields were tested, which was coupled with microscopic confirmation of causal pathogens. This step was to address adaptability to real-world situations, and hopefully this methodology will provide future researchers with a foundation to improve the dimensions of this work.

## Materials and methods

**Figure 1:**
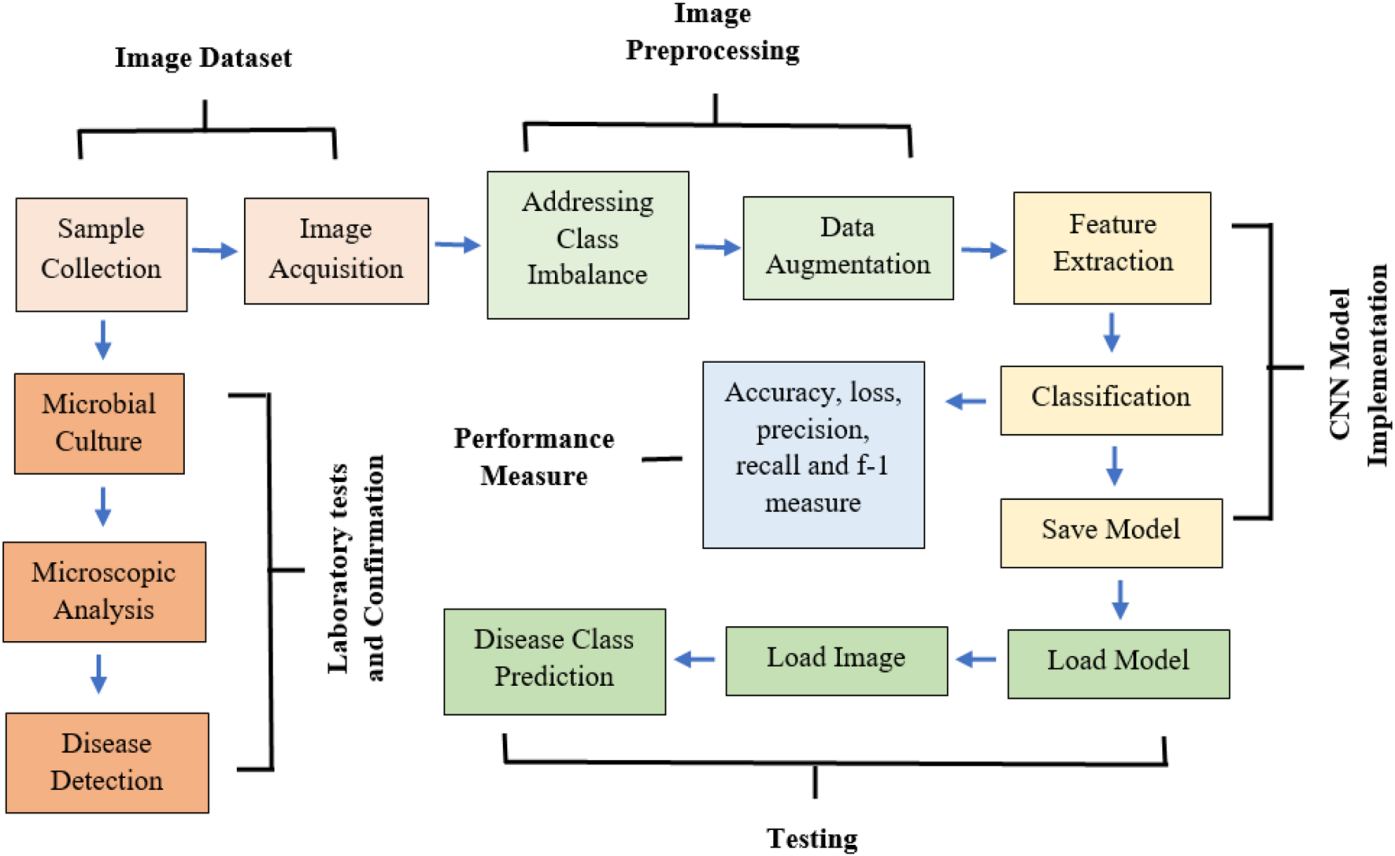
Block diagram of proposed approach.

The proposed approach for the development of CNN model is presented in block diagram in Fig. 1; The entirety of the proceedings was carried out in Python.

### Image dataset

The images in our dataset were acquired by collecting samples and capturing images directly from the different fields of Sylhet division: (i) Bangladesh Lakkatura Tea Garden, Sylhet, Bangladesh; ii) Malnicherra Tea State, Sylhet, Bangladesh; and (iii) Experimental Tea Garden, Department of Food Engineering and Tea Technology, SUST, Sylhet, Bangladesh. Tea leaves and images were collected in compliance with relevant guidelines provided by the authorities from each respected tea field, and formal permission was acquired. Throughout the data curation and disease identification process, four field experts were consulted under the supervision of Dr. Mainuddin Ahmed, a renowned entomologist in Bangladesh and former director of the Bangladesh Tea Research Institute (BTRI), and Professor Iftekhar Ahmad from the department of Food Engineering and Tea Technology, Shahjalal University of Science and Technology (SUST). The research did not involve any rare or endangered cultivars. The primary dataset was manually labeled using LabelImg, an open-source image annotation tool developed by TzuTa Lin from Label Studio. Images were captured using a Canon EOS 90D DSLR camera equipped with an 18-55 mm STM lens. The dataset comprised 3,330 images, with a distribution across classes as follows: 24.29% brown blight, 26.06% grey blight, 24.50% red rust, and 25.15% healthy leaves.

While many researchers use several accessible image datasets for research purposes, such as the “PlantVillage” image dataset, the APS image dataset, and so on^27^, our image dataset consisted wholly of primary material. It is considered that the megapixels of the camera and its orientation can affect the quality of the captured images^28^. To ensure the model could learn from detailed visual information, we prioritized capturing high-quality images for our primary dataset. This ensures the model can learn from detailed visual information, potentially leading to more accurate disease identification compared to secondary datasets with lower resolution or compressed images. This approach ensures the model extracts the most valuable information from each image for accurate disease detection. The samples were transported in a sterile polythene zipper bag, labeled by field experts, and brought to the laboratory for testing. The samples were stored at a room temperature of approximately 25 °C. Following are the signs and symptoms of the various commonly occurring tea diseases in Bangladesh:

- Brown blight: yellow–brown to chocolate-brown spots appear at the boundary area of leaves. Little black dots, similar to fruiting bodies, are produced on the affected part.
- Grey blight: little, slightly brown spots become visible on the upper surface of the leaves. Finally, the spot becomes dark brown with a slightly gray appearance at the core of the affected part.
- Red rust: tiny circular to semi-circular red-colored spots come into view on the surface of the infected leaf. Afterward, the small spots become steadily thicker.

In this paper, the dataset consists of pictures of four classes of RGB images. Some example images for each category of the dataset are shown in Table 1.

**Table 1:**
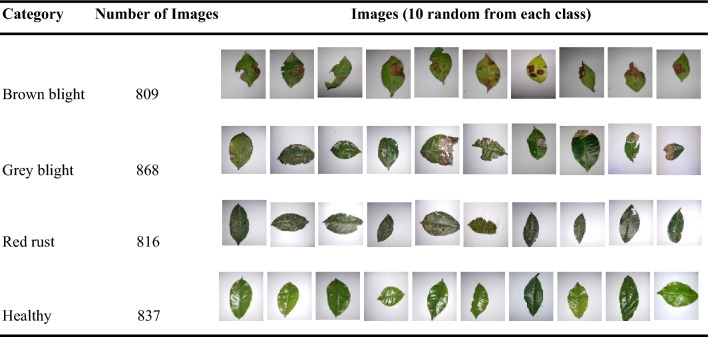
Sample images in dataset.

### Image preprocessing

**Figure 2:**
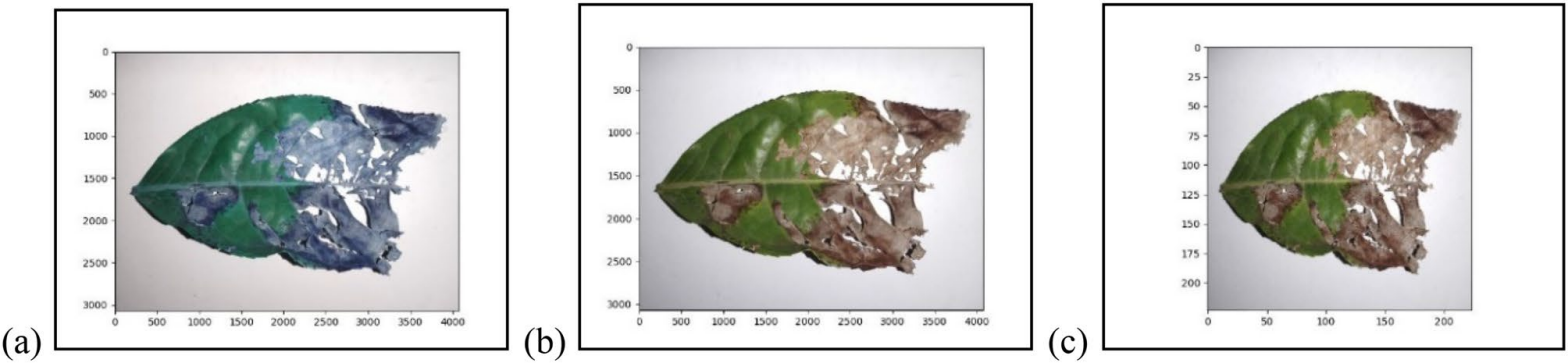
Image preprocessing (**a**) BGR format image; (**b**) RGB format image; (**c**) size reduction to 224 × 224.

The primary purpose of image pre-processing is to improve the quality of image and reduce the unwanted noise resulting from the presence of dust, shadows, and complex backgrounds. Preprocessing is a necessary step to make images suitable for further processing^29^. We read images using cv2.imread() function, and it converts images into BGR format. The images were transformed to RGB format, and the initial size of the images was large and dissimilar. To reduce processing time, the size of the images was reduced to 224 × 224, as shown in Fig. 2.

Overfitting is one of the inherent obstacles in machine learning that results from limitations of the data set, such as noise, a limited training set, classification complexity, etc.^30^. To improve the generalizability of our model and address the limitations of the dataset size, data augmentation techniques were performed using flow_from_directory(). As the pixel value of our images ranged from 0–255, we used rescale-1./255 to normalize it within 0 to 1 in order to reduce convergence and mitigate sensitivity due to lighting variations^31^. Furthermore, shear_range = 0.2 and zoom_range = 0.2 were applied for shearing transformation and randomly zooming images, respectively. Additionally, randomly flipping the images horizontally and vertically was also done.

Our dataset exhibits some degree of class imbalance, with class sizes ranging from 809 to 868. To address this imbalance and improve model performance for the minority class, we employed over-sampling, a technique that increases the representation of the minority class in the training data. We chose oversampling over under-sampling because our dataset size was clearly not overly large, and under-sampling could potentially discard valuable information^32^. Moreover, oversampling is reported to have performed well on comparable datasets such as ours^33^.

### CNN model implementation

**Figure 3:**
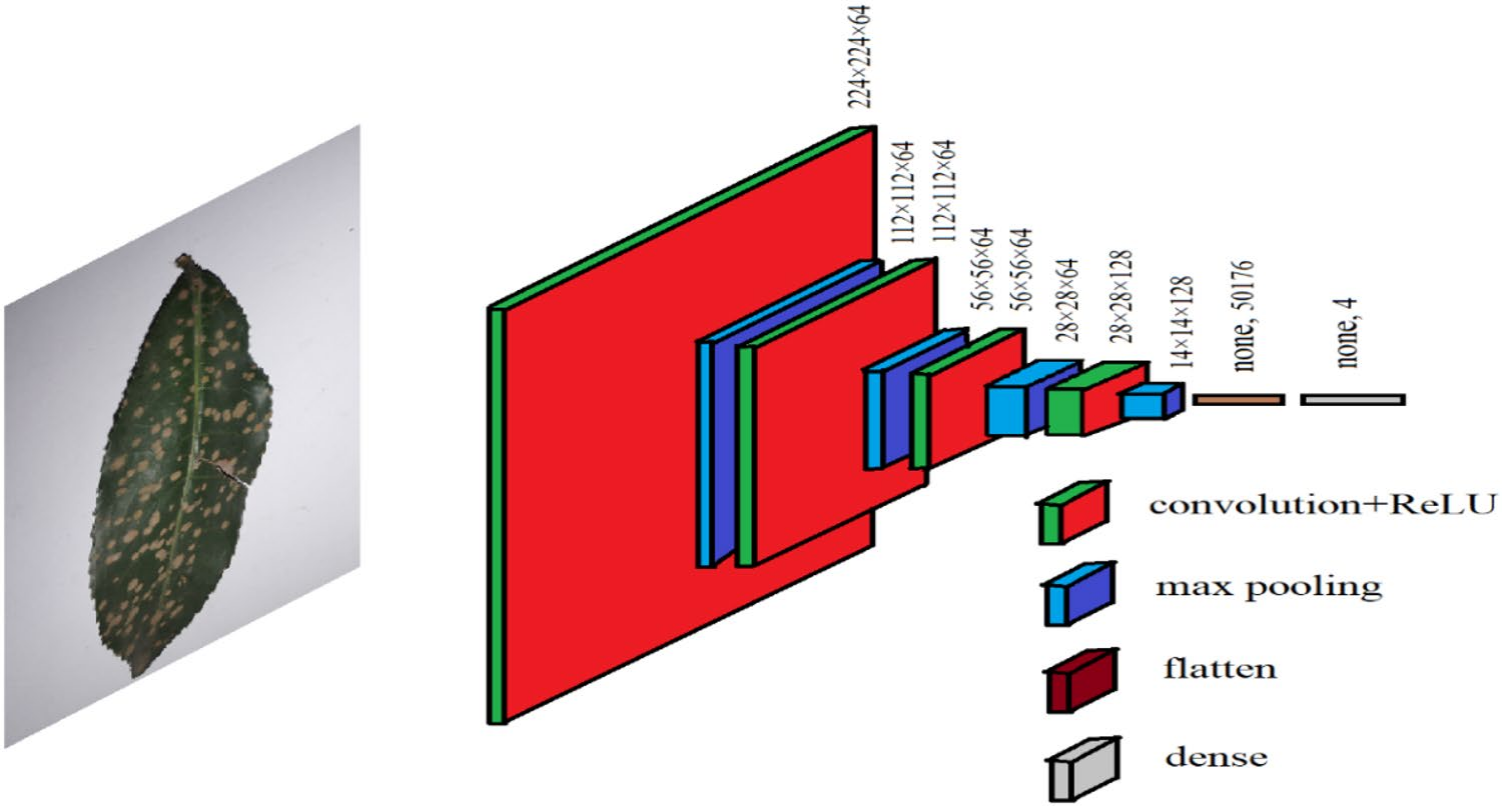
CNN Architecture for detection of tea leaf diseases.

As in the case of our limited dataset, CNN is a pragmatic starting point because it offers a good balance between efficiency, capability, and ease of use while maintaining high performance^34,35^. Owing to its simplicity and interpretability, a basic CNN was chosen to conduct this particular study, which can also be used as a gauge for comparison to other advanced models. A typical CNN model is fundamentally composed of convolution layers, activation layers, max-pooling layers, and fully connected layers. It is an architecture that receives a single vector of images as input and converts them into a series of multiple hidden layers. All the hidden layers consist of a collection of neurons, and every neuron is connected with all the neurons of previous layers. Firstly, the model extracts feature and then classifies the diseases. In this case, 70% of the total data were randomly selected for the purpose of training, and the other 30% were selected for testing. Various hyperparameter configurations were explored, which included different numbers of convolutional layers, filter sizes, and learning rates, and the architecture of Fig. 3 was found to have the best fit. The model was designed with four convolutional layers. The first three convolutional layers are composed of 64 filters of size 3 × 3. The last convolutional layer employed 128 filters of size 3 × 3. Four consecutive max-pooling layers were incorporated to further reduce dimensionality and extract features. The shear range and zoom range were 0.2. The batch size of the model was 16, while the learning rate was 0.001. The loss function was categorical cross-entropy. The images of the dataset were resized to fit into 224 × 224 dimensions, which were chosen as they were relatively close to the average size of all images. The padding of the model was kept the same for the study where stride was one. The Rectified Linear Unit (ReLu) function has been used as an activation function in each convolution layer. After the extraction of features, the final pixel matrix was flattened and transferred to the fully connected network as input. Softmax has been used in the fully connected layer as an activation function. Then the model was compiled with the Adam optimizer. We opted for the Adam optimizer due to its advantages in deep learning tasks, such as efficient handling of sparse gradients and good convergence behavior^36^. The tensor details at each layer of this architecture are presented in Table 2.

**Table 2:** Parameters obtained implemented CNN.

Layer (type)	Output Shape	Number of Parameters
conv2d (Conv2D)	(None, 224, 224, 64)	1792
max_pooling2d (MaxPooling2D)	(None, 112, 112, 64)	0
conv2d_1 (Conv2D)	(None, 112, 112, 64)	36,928
max_pooling2d_1 (MaxPooling2D)	(None, 56, 56, 64)	0
conv2d_2 (Conv2D)	(None, 56, 56, 64)	36,928
max_pooling2d_2 (MaxPooling2D)	(None, 28, 28, 64)	0
conv2d_3 (Conv2D)	(None, 28, 28, 128)	73,856
max_pooling2d_3 (MaxPooling2D)	(None, 14, 14, 128)	0
flatten (Flatten)	(None, 50, 176)	0
dense (Dense)	(None, 4)	100,356

**Figure 4:**
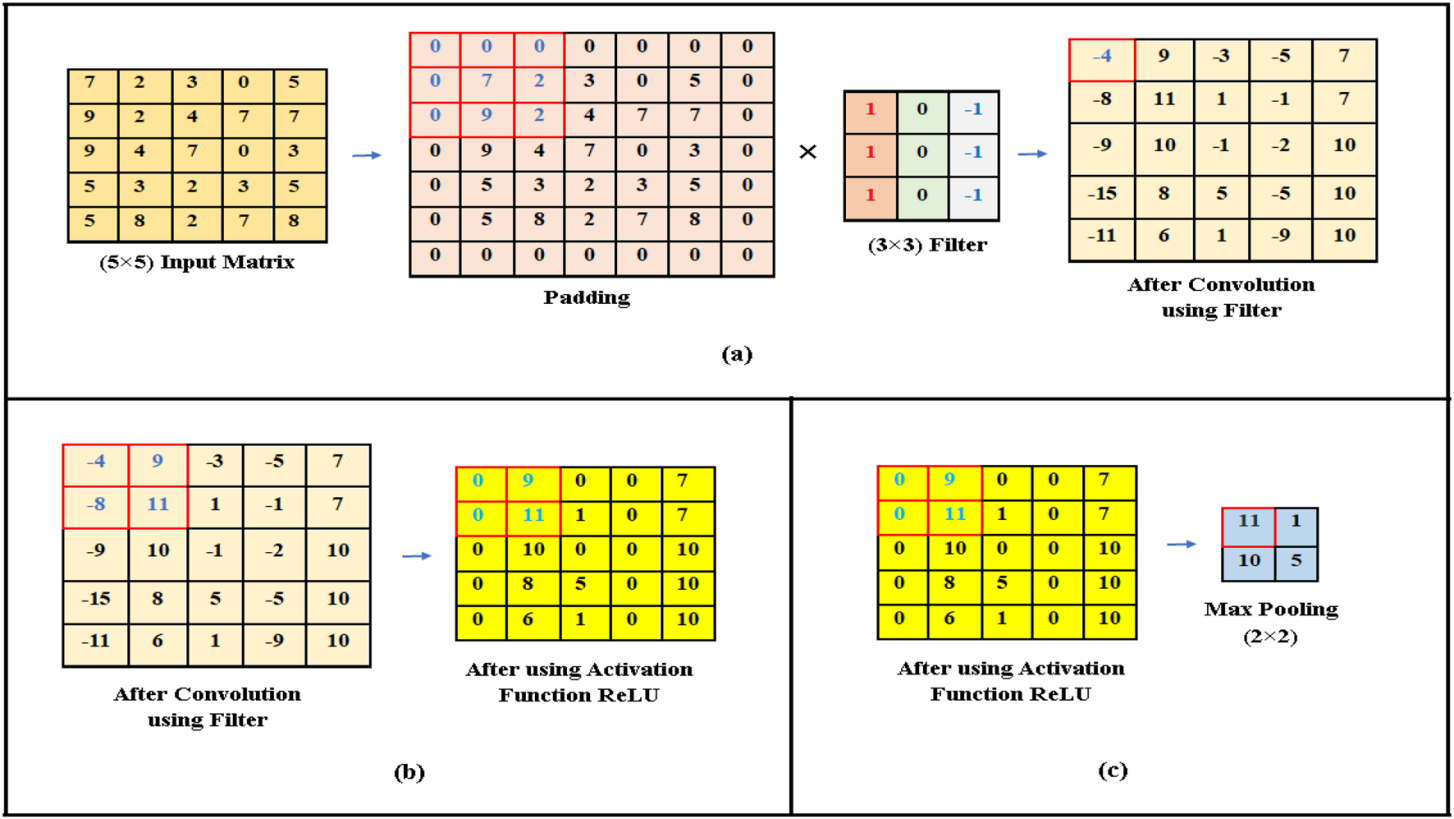
CNN Operations (**a**) convolution operation (5 × 5 input image) and (3 × 3 filter); (**b**) ReLu operation for 5 × 5 matrix; (**c**) max pooling operation for 2 × 2 filters.

The architecture of the proposed research work was presented in Fig. 3;

*Convolution Layer*: A convolutional layer is fundamental to the CNN model to perform feature extraction. The model was designed with 4 convolutional layers. Convolution layers take RGB images as input from a specific folder via given instructions and send them as output to another layer for further processing. After receiving input, the layer reads pixels as values to produce feature maps. The output calculation was done by summation of an element-by-element multiplication of the input pixel value with the filter value. An example of a convolution operation for a 5 × 5 input image and a 3 × 3 filter is presented in Fig. 4a.

*Activation Layer*: The architecture utilized ReLU as the activation layer after each convolutional layer. The model was designed with four layers of ReLU activation functions. By incorporating ReLU, the model enhanced its non-linear properties while preserving positive values from the convolutional layer. It also changes all negative values to zero. The equation will be: f(x) = max (0, x). An example of a ReLU operation with a 5 × 5 matrix is presented in Fig. 4b.

*Pooling Layer*: 4 Max-pooling layers were used in this model, where the maximum value taken via max-pooling from every sub-region was bound via filter. An example of a pooling operation is presented in Fig. 4c.

*Fully connected layer*: These were used in the model to detect very high levels of features, and those layers connect to the previous layers where neurons transfer vectors of input by a weight matrix. The fully connected layer output was one-dimensional via flattening the output of the pooling layer. The classification of diseases is performed in this fully connected layer.

### Metrics of evaluation of the model’s efficiency

The efficiency of the CNN model was estimated by precision, recall, f1 score, and accuracy. These metrics are commonly employed to determine the performance of a machine learning algorithm. Precision, shown in Eq. (1), is the estimation of true positives among all the positive predictions made by the model. Recall, or true positive rate (Eq. 2), focuses on how well the model is capable of identifying all the positives. The F1 score is defined as a metric that merges precision and recall into a single value, as presented in Eq. (3). The range of the metrics is from 0 to 1, where a higher score indicates better performance. Moreover, the accuracy of each individual class was also measured. Accuracy is a metric used to measure how well a model can correctly identify objects within an image. It's calculated by dividing the number of correct predictions by the total number of predictions made. Following are the equations for these performance metrics:

1 $$Precision=\frac{True\,Positives}{True\,Positives+False\,Positives}$$

2 $$Recall=\frac{True\,Positives}{True\,Positives+False\,Negatives}$$

3 $$F1 Score=2\times \frac{Precision \times Recall}{Precision+Recall}$$

4 $$Accuracy=\frac{True\,Negative+True\,Positive}{True\,Negative+False\,Positive+True\,Positive+False\,Negative}\times 100$$

### Method of laboratory tests

To identify the pathogens, a total of 120 symptomatic leaves from three diseased classes and 40 healthy leaves were brought into the laboratory for microscopic examination in order to observe the pathogen's morphological characteristics. The healthy leaves were observed with the naked eye. The surface of the symptomatic leaves was washed with 2% Sodium Hypochlorite (NaOCl) for 2 min, rinsed in sterilized distilled water. The infected part of the leaves was cut with a sterilized blade, and the smaller portions were further cut into smaller pieces. Afterwards, Potato Dextrose Agar solution was made by following the manufacturer’s ratio (39 gm/1000 ml). Then the suspension was heated and stirred on a magnetic stirrer to dissolve the agar solution. Dissolved media was autoclaved at 15 lbs. pressure and 121 °C for 15 min. After sterilization, dissolved PDA was poured into Petri dishes, and it was allowed to cool down until it solidified. 250 µl samples from 10^–1^ and 10^–2^ dilutions were poured into different petri dishes using micro pipet. The petri dishes were wrapped in parafilm and incubated in a chamber at 25 °C for 6 days. For microscopic observation, a clean slide was taken, 1 drop of lactophenol cotton blue was added to the slide via a plastic dropper, a small portion of culture was taken from petri dishes via sterilized forceps and put into the cotton blue drop, smeared by fungal or algal loop, and the prepared slide was observed under a binocular microscope.

## Results and discussion

Image dataset preparation, model implementation, and performance metrics were included in this work. A large dataset of primary images was built. We focused on a model based on a convolutional neural network that was implemented with 40 epochs, where epochs are defined as the number of times the entire training set is passed through the network, and training, testing, and validation were applied. We employed early stopping to prevent overfitting by monitoring validation accuracy and loss during training. Training was stopped after 40 epochs as the gap between validation accuracy and training accuracy plateaued, and validation loss did not exhibit significant improvement. It took approximately 13 h for training.

### Simulation environment

The proposed CNN model was developed using numpy, pandas, os, re, shutil, PIL, matplotlib, tdqm, cv2, tensorflow, and Keras libraries. The model was implemented by an Anaconda environment (Jupyter Notebook) on a laptop with Windows 10 Pro, 8 GB of random-access memory, an Intel Core i5 Central Processing Unit, a 256 GB solid-state drive, and a 64-bit operating system.

### Performance measure

**Figure 5:**
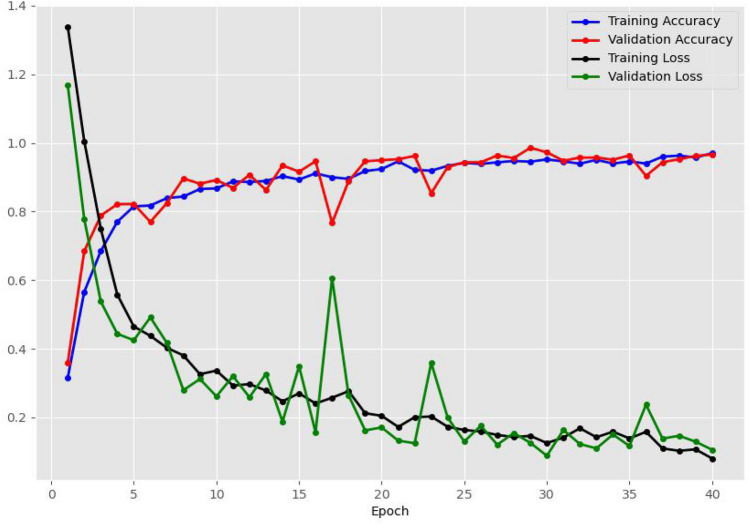
Accuracy and loss after CNN model implementation.

The model’s performance was measured in various ways. The loss of the model was minimized by the Adaptive Moment Estimation (ADAM) optimizer. It is observed that the final training accuracy of the model is 97.02% and the validation accuracy is 96.65%. Accuracy and loss after implementation of the model are graphically presented in Fig. 5.

**Figure 6:**
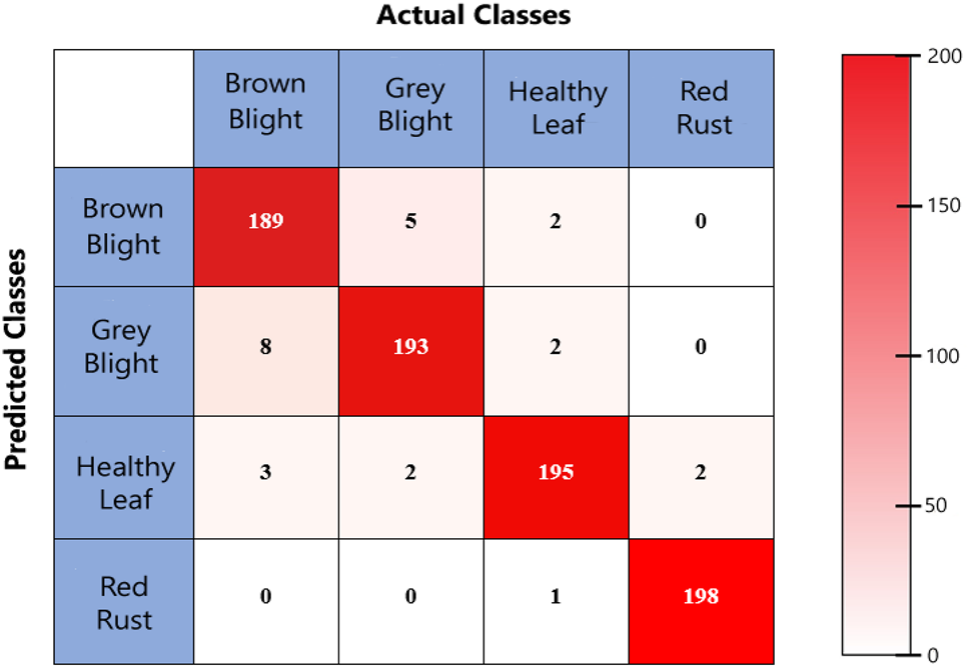
Confusion matrix of the proposed model.

A notable increment in both training and validation accuracy was observed during the first five iterations of training, accompanied by a simultaneous reduction in associated training and validation losses**.** From epochs 5 to 40, the accuracy and loss fluctuated gradually, and the validation accuracy swiftly increased from epoch 17. The confusion metrics of our proposed model have been designed based on the samples that are unknown to the model dataset. Thakur et al. evaluated five different publicly available datasets of different species of crops^37^, while another study^38^ showed confusion metrics for different 10 classes to evaluate different performances. The classification performance of their model was 90% to 99%. In the confusion matrices presented in Fig. 6, the number of true positives, false positives, true negatives, and false negatives was analyzed.

**Figure 7:**
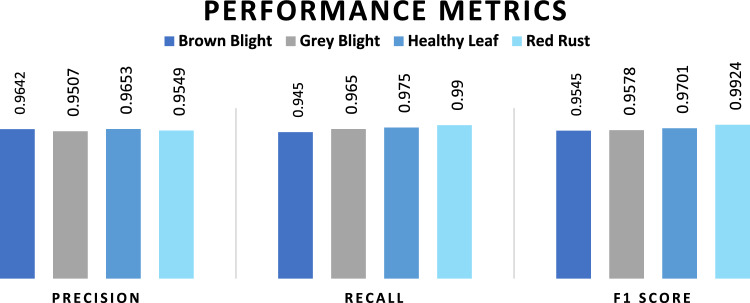
Performance evaluation of proposed CNN model.

Performance metrics such as precision, recall, and F1-value were identified in the model because they are indicative of its strengths and limitations. First of all, various performance evaluation metrics were calculated for brown blight disease, where the true positive number is 189, the false positive number is 8, and the false negative number is 11. True positivity means both predicted and actual values are positive. On the other hand, false positive implies predicted values are positive, but the actual values are negative. Moreover, false negatives mean both predicted and actual values are negative. The results of precision, recall, and F1-value of the proposed model are graphically represented in Fig. 7.

We got 0.9642 of precision, 0.945 of recall, and 0.9545 of f1-score for brown blight disease. For grey blight disease, the true positive number is 193, the false positive number is 10, and the false negative number is 7. This resulted in 0.9507 of precision, 0.965 of recall, and 0.9578 of f1-score for grey blight. Similarly, for the healthy leaf class, the precision, recall, and f1-value were 0.9653, 0.975, and 0.9699, respectively. Finally, for red rust disease, 0.9549 of precision, 0.99 of recall, and 0.9924 of f1-score have been found for the proposed model. A recent, comparable study on tomato leaf disease detection found that the red rust class had 0.975 precision and a 0.982 f1 score in their study by VGG-16, which was highest, while lowest performance was observed in the brown blight class^26^.

**Figure 8:**
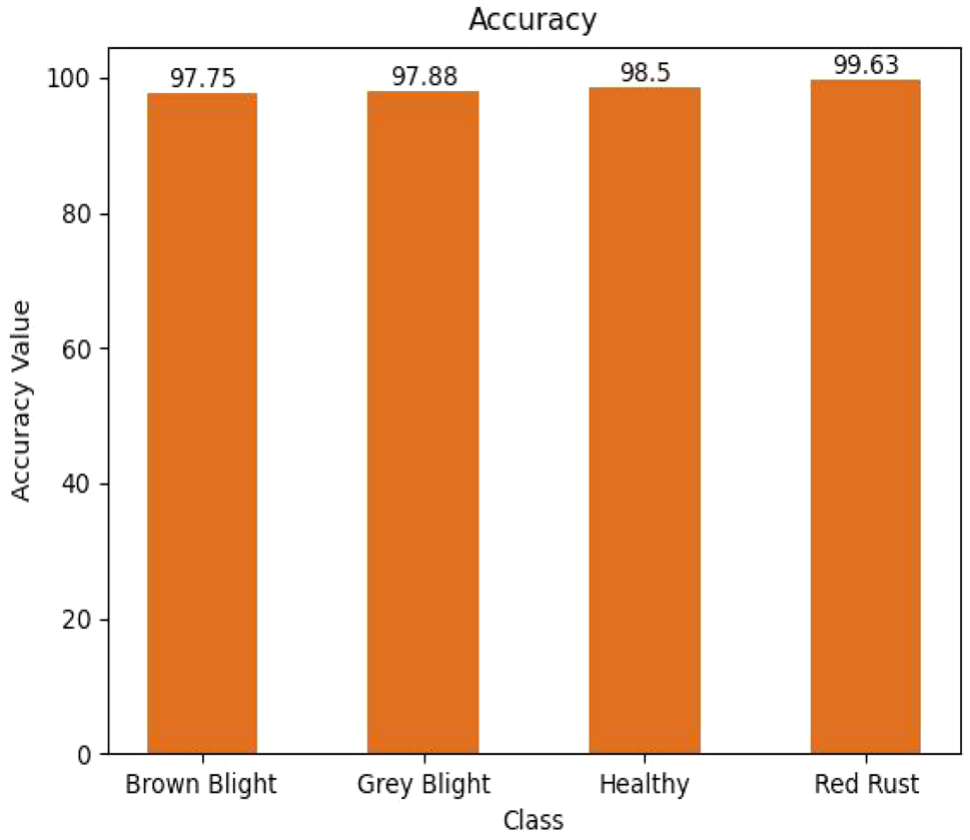
Accuracy level of each class.

The accuracy of each disease class was also evaluated and presented in graphical form in Fig. 8. Our model achieved exceptional performance across all disease classes, with individual accuracies ranging from 97.75% to 99.63%. This degree of consistency demonstrates the model's ability to avoid overfitting and to identify various tea leaf diseases.

The performance of the proposed CNN model was compared against the methods reported in the literature according to accuracy presented in Table 3.

**Table 3:** Performance comparison of proposed CNN model with other existing models.

Sl. No	Source	Collection of data	Work on	Lab test	Size of dataset	Methods	Class	Accuracy (%)
01	^39^	Secondary	Tomato diseases	No	3000 images	CNN	6	76
02	^40^	Primary, from field	apple leaf disease	No	5201 images	CNN	10	81.09
03	^41^	secondary, from CommIT	tea plant diseases	No	4727 images	GoogLeNet	4	78.65
						Xception	4	87.10
						Inception-ResNet-v2	4	87.95
04	^42^	Secondary, from plant village dataset	Tomato leaf disease	No	18,160 images	CNN	10	98.4
05	^43^	Secondary, from plant village dataset	Tomato leaf diseases	No	50,000 images	CNN	14	91.2
06	^44^	Primary, from field	Tea leaf diseases	No	144 images	CNN	4	92.5
07	^45^	primary, from Heilongjiang Academy of Land Reclamation Sciences, China	rice diseases	No	500 images	CNN	10	95
08	^46^	Secondary, Kaggle official website	Apple, cherry and corn diseases	No	22,427 images	ResNet50	10	95.1
						DenseNet169	10	99
						EfficientNet	10	98.4
						FL-EfficientNet	10	99.7
09	Proposed approach	Primary, collected from different tea fields of Sylhet division	Tea leaf diseases	Yes	3330 images	CNN	4	96.65

*Confirmation with Laboratory Tests*: To the best of our knowledge, no previous automated disease identification study conducted confirmation of disease-causing pathogens to solidify the predictions. For the purpose of cross-validation with laboratory tests and to ensure the generalizability of our model to real-world scenarios, we collected an independent validation set of 160 unknown leaf samples directly from the tea plantations. This approach ensured the samples originated from the same geographical location and potentially shared similar environmental factors and tea plant varieties as the original dataset. To ensure our unknown samples reflected a realistic mix of disease presence, we implemented a stratified random sampling approach within the tea plantations. This involved dividing the entire collection of tea leaves into smaller groups based on the specific disease they exhibited (healthy, brown blight, grey blight, red rust). We then randomly selected samples from each of these disease groups, aiming to collect approximately 40 samples for each category. Sourcing unknown samples from the same plantations ensured consistency in the ecological context, including disease prevalence specific to that region. While these leaf samples weren't part of the original dataset, they were randomly collected to provide a complementary layer of validation to the model's predictions in a real-world context. These validation samples were observed in the laboratory with microbial culture media and microscopic analysis to compare causal pathogens and further ensure the specificity of the predictions. Table 4 presents the prediction results of the model compared to the results of laboratory tests.

**Table 4:**
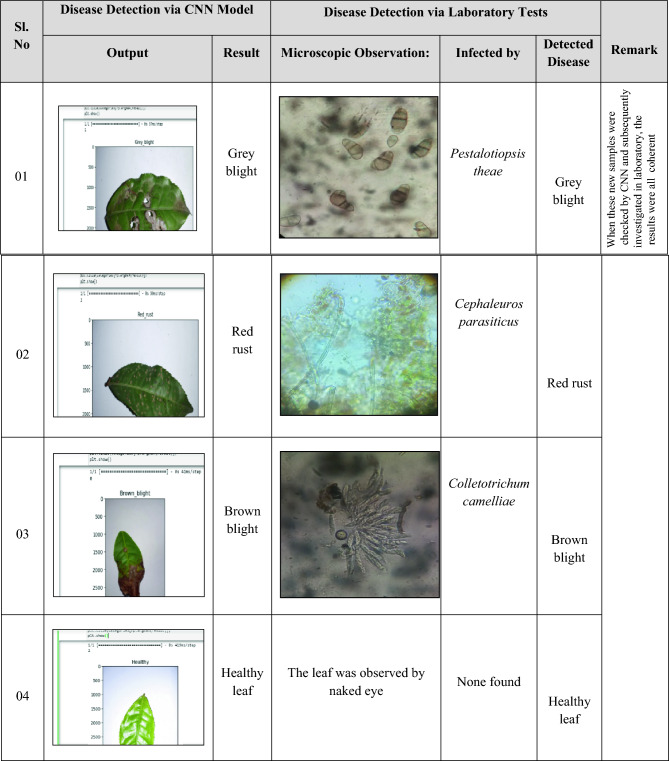
Cross-checking CNN model's prediction with laboratory test.

For the first sample, it was found that the leaf had been infected by *Pestalotiopsis theae,* showing grey blight symptoms, which is concordant with previous findings^34^. Then the picture of the leaf was taken and checked by this proposed CNN model, and the results of the model and laboratory test are similar. For samples 2 and 3, it was observed by laboratory tests that the samples were infected by *Cephaleuros parasiticus* and *Colletotrichum camelliae,* the causal agents for red rust disease and brown blight disease, respectively, which was identical to the detected disease by our model. On the other hand, the leaf of sample 4 was healthy and exhibited no pathogenic symptoms, which was predicted as a healthy leaf by our model. In every case of cross-validation, the results of both laboratory tests and the CNN model were found to be invariably coherent.

## Conclusion and future perspective

In the context of tea production, timely identification and management of diseases are critical for optimizing yields during planting and harvesting. In developing countries like Bangladesh, where computational technology is increasingly accessible, improved disease detection systems for tea estates hold significant economic potential. This study investigated the application of a CNN model for detecting and identifying various tea leaf diseases. The proposed CNN model successfully distinguished three distinct disease classes and differentiated between healthy and diseased leaves. The accuracy of each disease class, namely brown blight, grey blight, healthy leaf, and red rust, was found to be 97.75%, 97.88%, 98.5%, and 99.63%, respectively. This represents strong class-specific performance. The model also achieved high overall accuracy (96.65%), along with strong recall, precision, and f1 scores ranging from 0.94 to 0.99. Notably, these results surpass those reported for previously employed CNN models in the discussion section. Additionally, cross-validation with laboratory tests yielded a perfect match between the model's predictions and the presence of causal pathogens confirmed through microscopic examination of all samples.

However, the current study has its limitations. Future research could explore more sophisticated oversampling techniques like Synthetic Minority Oversampling (SMOTE), advanced CNN architectures like ResNet, VGG, or DenseNet, potentially fine-tuned with transfer learning techniques, to investigate their adaptability and performance on the task of tea leaf disease classification. Additionally, dataset expansion remains a key area for improvement. Future work should focus on collecting a wider variety of diseased tea leaf samples encompassing diverse cultivars, fertility stages, and shooting angles within field settings. By incorporating microscopic confirmation of disease presence alongside image data, researchers can further strengthen the model's accuracy and reliability.

## Data Availability

The datasets generated and analyzed in the course of the current study are not openly accessible; however, interested parties may obtain them from the corresponding author upon submitting a reasonable request.

## References

[1] Nasir T, Shamsuddoha M (2011). Tea productions, consumptions and exports: Bangladesh perspective. Int. J. Educ. Res. Technol..

[2] Hayat K (2015). Tea and its consumption: Benefits and risks. Crit. Rev. Food Sci. Nutr..

[3] Hu G (2021). Detection and severity analysis of tea leaf blight based on deep learning. Comput. Electr. Eng..

[4] Dutta P (2008). Red rust: An emerging concern. Two Bud.

[5] Pandey AK (2021). How the global tea industry copes with fungal diseases–challenges and opportunities. Plant Disease.

[6] Arulpragasam, P., Addaickan, S. & Kulatunga, S. *Recent developments in the chemical control of blister blight leaf disease of tea-effectiveness of EBI fungicides* (1987).

[7] Gulati A (1993). Economic yield losses caused by Exobasidium vexans in tea plantations. Indian Phytopathol..

[8] Radhakrishnan, B. & Baby, U. *Economic threshold level for blister blight of tea.* Planters Chronicle 4 (2005).

[9] Keith, L., Ko, W.-H. & Sato, D. M. *Identification guide for diseases of tea (Camellia sinensis)* (2006).

[10] Ponmurugan P, Saravanan D, Ramya M (2010). Culture and biochemical analysis of a tea Algal pathogen, *Cephaleuros*
*parasiticus* 1. J. Phycol..

[11] Ponmurugan P (2009). Studies on *Cephaleuros*
*parasiticus* Karst, a pathogenic alga causing red rust disease in tea plantations. J. Plant. Crops.

[12] Devaraj, A., et al. *Identification of plant disease using image processing technique*. In *2019 International Conference on Communication and Signal Processing (ICCSP)*. 2019. IEEE.

[13] Ghaiwat SN, Arora P (2014). Detection and classification of plant leaf diseases using image processing techniques: A review. Int. J. Recent Adv. Eng. Technol..

[14] Patil JK, Kumar R (2011). Color feature extraction of tomato leaf diseases. Int. J. Eng. Trends Technol..

[15] Rathod AN, Tanawal B, Shah V (2013). Image processing techniques for detection of leaf disease. Int. J. Adv. Res. Comput. Sci. Softw. Eng..

[16] Chen Y (2017). Characterization, pathogenicity, and phylogenetic analyses of Colletotrichum species associated with brown blight disease on Camellia sinensis in China. Plant Dis..

[17] Lu Q (2018). Differences in the characteristics and pathogenicity of *Colletotrichum*
*camelliae* and *C.*
*fructicola* isolated from the tea plant [*Camellia*
*sinensis* (L.) O Kuntze]. Front. Microbiol..

[18] Sen S (2020). Blister blight a threatened problem in tea industry: A review. J. King Saud Univ. Sci..

[19] Huq M, Ali M, Islam M (2010). Efficacy of muriate of potash and foliar spray with fungtcides to control red rust disease (*Cephaleurous*
*parasiticus*) of tea. Bangladesh J. Agric. Res..

[20] Chen J, Liu Q, Gao L (2019). Visual tea leaf disease recognition using a convolutional neural network model. Symmetry.

[21] Al Bashish D, Braik M, Bani-Ahmad S (2011). Detection and classification of leaf diseases using K-means-based segmentation and neural-networks-based classification. Inf. Technol. J..

[22] Ashwinkumar S (2022). Automated plant leaf disease detection and classification using optimal MobileNet based convolutional neural networks. Mater. Today Proc..

[23] Ramcharan A (2019). A mobile-based deep learning model for cassava disease diagnosis. Front. Plant Sci..

[24] Rangarajan AK, Purushothaman R, Ramesh A (2018). Tomato crop disease classification using pre-trained deep learning algorithm. Procedia Comput. Sci..

[25] DeChant C (2017). Automated identification of northern leaf blight-infected maize plants from field imagery using deep learning. Phytopathology.

[26] Kibriya, H., et al. *Tomato leaf disease detection using convolution neural network*. In *2021 International Bhurban Conference on Applied Sciences and Technologies (IBCAST)*. 2021. IEEE.

[27] Mohanty SP, Hughes DP, Salathé M (2016). Using deep learning for image-based plant disease detection. Front. Plant Sci..

[28] Triantaphillidou S, Smejkal J, Fry E (2020). Studies on the effect of megapixel sensor resolution on displayed image quality and relevant metrics. Electronic Imaging.

[29] Bera, T., et al. *A survey on rice plant disease identification using image processing and data mining techniques*. In *Emerging Technologies in Data Mining and Information Security: Proceedings of IEMIS 2018, Volume 3*. Springer (2019).

[30] Ying, X. An overview of overfitting and its solutions. In *Journal of physics: Conference series*. 2019. IOP Publishing.

[31] Sendjasni, A., Traparic, D. & Larabi, M.-C. *Investigating normalization methods for CNN-based image quality assessment*. In *2022 IEEE International Conference on Image Processing (ICIP)*. IEEE (2022).

[32] Batista GE, Prati RC, Monard MC (2004). A study of the behavior of several methods for balancing machine learning training data. ACM SIGKDD Explor. Newsl..

[33] Bach M (2017). The study of under-and over-sampling methods’ utility in analysis of highly imbalanced data on osteoporosis. Inf. Sci..

[34] Tariqul Islam, M. & Tusher, A. N. *Automatic detection of Grape, Potato and Strawberry Leaf Diseases using CNN and image processing*. In *Data Engineering for Smart Systems: Proceedings of SSIC 2021*. 2022. Springer.

[35] Paymode, A. S., Magar, S. P. & Malode, V. B. Tomato leaf disease detection and classification using convolution neural network. In *2021 International Conference on Emerging Smart Computing and Informatics (ESCI)*. IEEE (2021).

[36] Ogundokun, R.O., et al. Improved CNN based on batch normalization and adam optimizer. In *International Conference on Computational Science and Its Applications*. Springer (2022).

[37] Thakur PS, Sheorey T, Ojha A (2023). VGG-ICNN: A Lightweight CNN model for crop disease identification. Multimedia Tools Appl..

[38] Gonzalez-Huitron V (2021). Disease detection in tomato leaves via CNN with lightweight architectures implemented in Raspberry Pi 4. Comput. Electron. Agric..

[39] Ferdouse Ahmed Foysal, M., et al. A novel approach for tomato diseases classification based on deep convolutional neural networks. In *Proceedings of International Joint Conference on Computational Intelligence: IJCCI 2018*. Springer (2020).

[40] Khan AI (2022). Deep diagnosis: A real-time apple leaf disease detection system based on deep learning. Comput. Electron. Agric..

[41] Krisnandi D (2019). Diseases classification for tea plant using concatenated convolution neural network. CommIT (Commun. Inf. Technol.) J..

[42] Agarwal M (2020). ToLeD: Tomato leaf disease detection using convolution neural network. Procedia Comput. Sci..

[43] Agarwal M, Gupta SK, Biswas K (2020). Development of efficient CNN model for Tomato crop disease identification. Sustain. Comput. Inform. Syst..

[44] Hu G (2019). Identification of tea leaf diseases by using an improved deep convolutional neural network. Sustain. Comput. Inform. Syst..

[45] Lu Y (2017). Identification of rice diseases using deep convolutional neural networks. Neurocomputing.

[46] Sun X (2022). Research on plant disease identification based on CNN. Cognit. Robot..

